# Supplementary material for: H3K27ac nucleosomes facilitate HMGN localization at regulatory sites to modulate chromatin binding of transcription factors
Source: Commun Biol. 2022 Feb 23;5:159. doi: 10.1038/s42003-022-03099-0 (PMC8866397; doi:10.1038/s42003-022-03099-0)
Supplement: Supplementary file 1 — Supplemental Information [file 42003_2022_3099_MOESM1_ESM.pdf]

Supplementary Information

H3K27ac Nucleosomes Facilitate HMGN Localization at Regulatory Sites to Modulate Chromatin Binding of Transcription Factors

Shaofei Zhang[1#], Yuri Postnikov[1#], Alexei Lobanov[2,3], Takashi Furusawa[1], Tao Deng[1,4], and Michael Bustin[1*]

[1]Protein Section, Laboratory of Metabolism, Center for Cancer Research, National Cancer Institute, [2]CCR Collaborative Bioinformatics Resource, Center for Cancer Research, National Cancer Institute, National Institutes of Health, Bethesda, MD, USA, [3]Advanced Biomedical Computational Science, Frederick National Laboratory for Cancer Research, MD, USA, [4]Stem Cell Translation Laboratory, NCATS, National Institutes of Health, 9800 Medical Center Drive, Rockville, MD 20850

Keywords: Epigenetic Regulation, chromatin, HMGN, transcription factor binding

Contains:

11 Supplementary Figures

1 Supplementary Table

1 Supplementary methods file

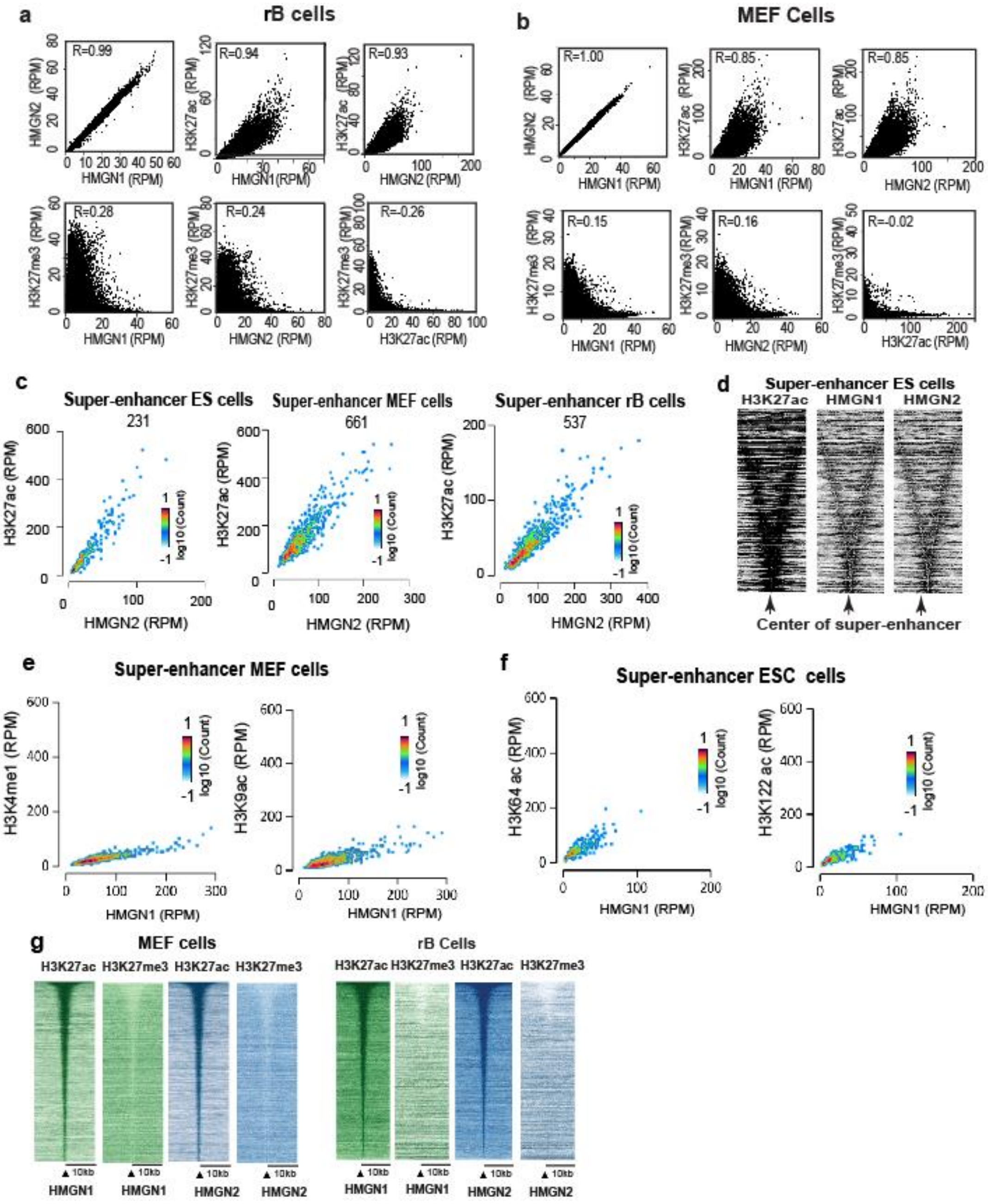

Supplementary Figure 1. HMGNs localize to H3K27ac nucleosomes (a) Scatter plot showing a direct correlation between HMGN occupancy levels and H3K27ac, but not H3K27me3 in rBs (**b)** Scatter plot showing a direct correlation between HMGN occupancy levels and H3K27ac, but not H3K27me3 in MEFs (**c)** Scatter plot showing direct correlation between HMGN2 and H3K27ac occupancy levels at the specific super-enhancers of ESCs, MEFs and rB cells. The number of super-enhancers in each cell-type is shown. (**d)** Heat map showing correlation between the occupancy levels of HMGN1, HMGN2 and H3K27ac within the ESCs super-enhancers. (**e)** Scatter plot showing the correlation between HMGN1 occupancy levels and H3K4me1 and H3K9ac at MEF super enhancers. (**f)** Scatter plot showing correlation between HMGN1 occupancy levels and H3K64ac and H3K122ac at mouse ESCs super-enhancers. **(g)** Heat map showing localization of the H3K27ac, but not of the H3K27me3 signal at chromatin sites containing either HMGN1 or HMGN2 in MEFs and rB cells.

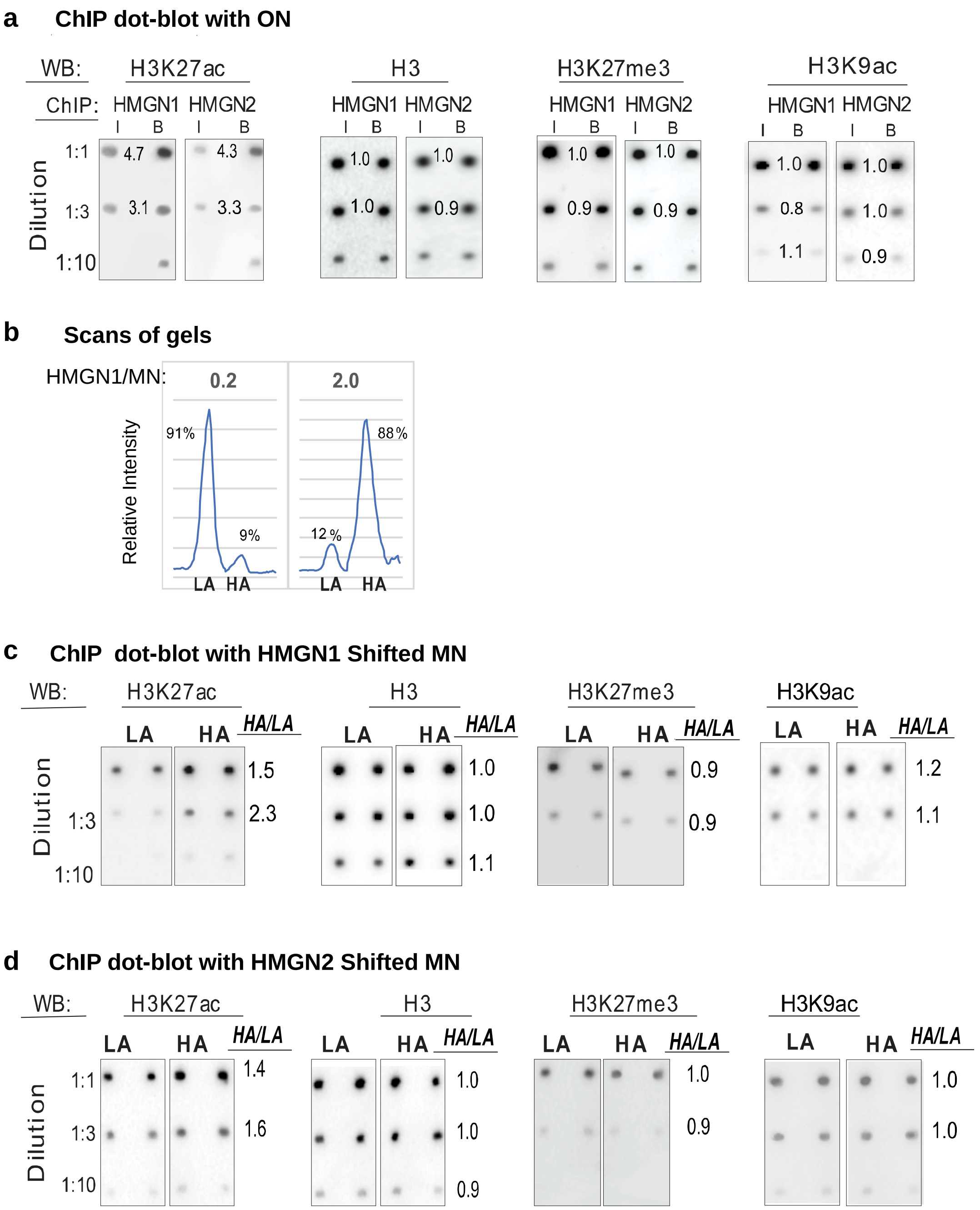

Supplementary Figure 2. **Preferential binding of HMGNs to H3K27ac nucleosomes**. **(a)** ChIP dot-blot Western with ON. HMGN1 or HMGN2 added to ON fraction (HMGN:histone H3 = 1:50), the complexes immunoprecipitated, the histone H3 from input (I) and HMGN-bound **(B)** ON purified, applied at 3 separate dilutions to PVD membranes, the membranes subjected to Western analysis with the antibodies indicated at top of the panels, and the intensity of the signal quantified. The numbers between the dots indicate the B/I ratio. Note the high B/I ratio for H3K27ac but not for H3K27me3, H3K9ac or H3 which serves as a loading control. Two strips were tested for each experiment. **(b)** Scans of selected lanes from Fig. 2C showing the percent of nucleosomes designated as either HA or LA. **(c,d)** Dot-blot Western analysis of LA and HA particles. The relative levels of H3K27ac in purified H3 determined as described in panel A except that the mobility shift was done on an HMGN:MN =2. Note the relative high HA/LA ratio for H3K27ac, but not for the three controls. Each strip contains duplicate samples; two strips were tested for each experiment.

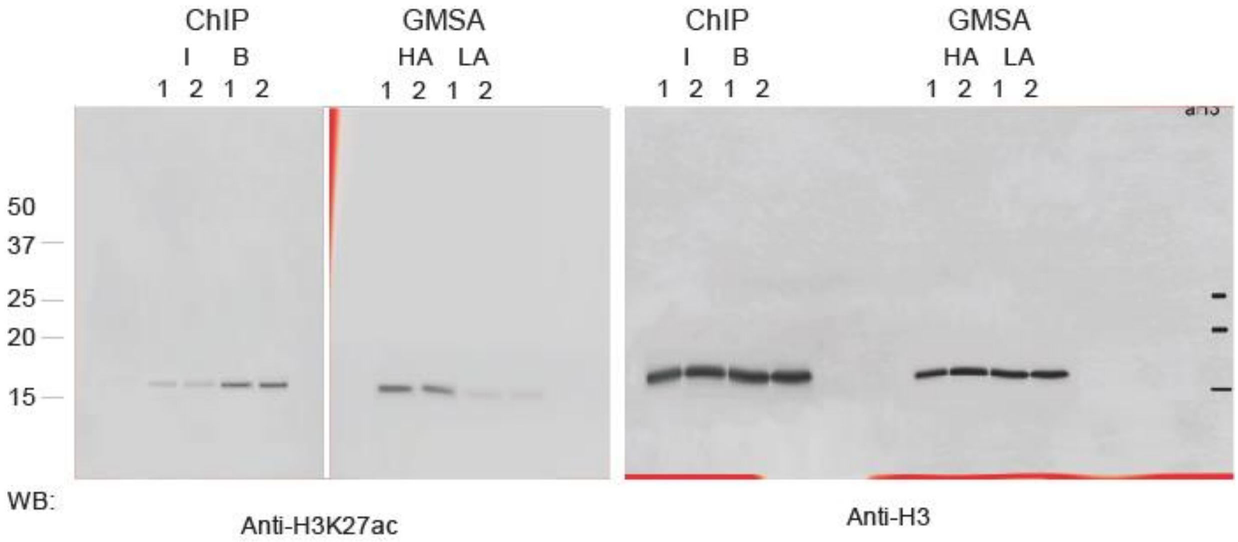

Supplementary Figure 3. Uncropped Western blot image with the size markers for Fig. 2b,d. Western analysis of total ON Input (I) and bound HMGN1 immunoprecipitated ChIP ON (B). Western analysis of high affinity (HA) and low affinity (LA) MN for gel mobility shift assay (B). Antibody for Western used: H3K27ac and histone H3.

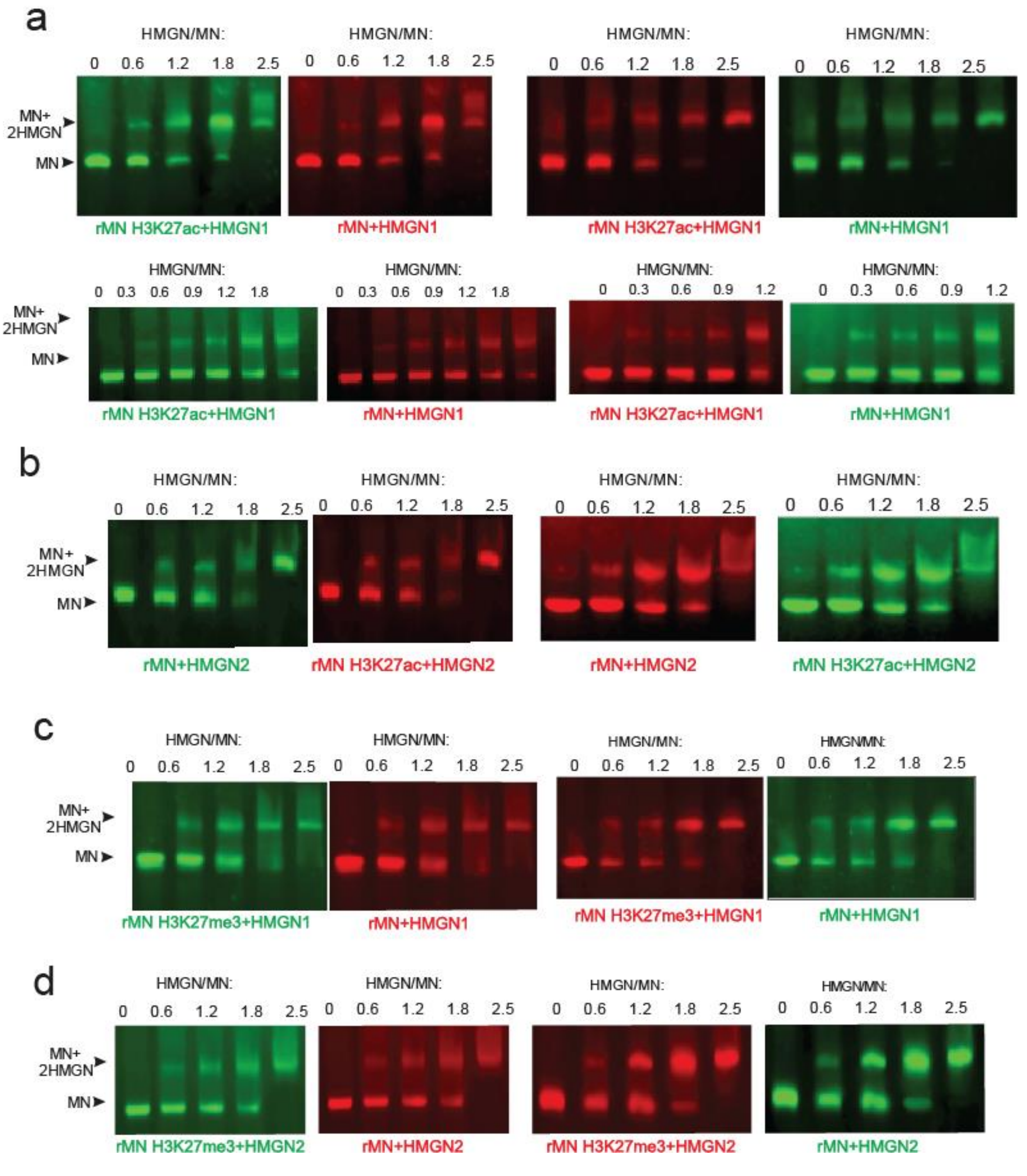

Supplementary Figure 4. Original two-color scans of nucleosome mobility shift assays shown in Fig. 2g. (a,b) Two color gel mobility shift assays of recombinant mononucleosomes (rMN). A mix of equal amounts of fluorescently Alexa 488 labeled rMN (green) and Alexa 647 labeled rMNH3K27ac (red) and with reverse staining were incubated with various amounts of HMGN1 or 2, the mixture fractionated on native polyacrylamide gels, and the gels scanned to visualize and quantify either the red or green fluorescence. **(c,d)** Same is for rMN H3K27me3.

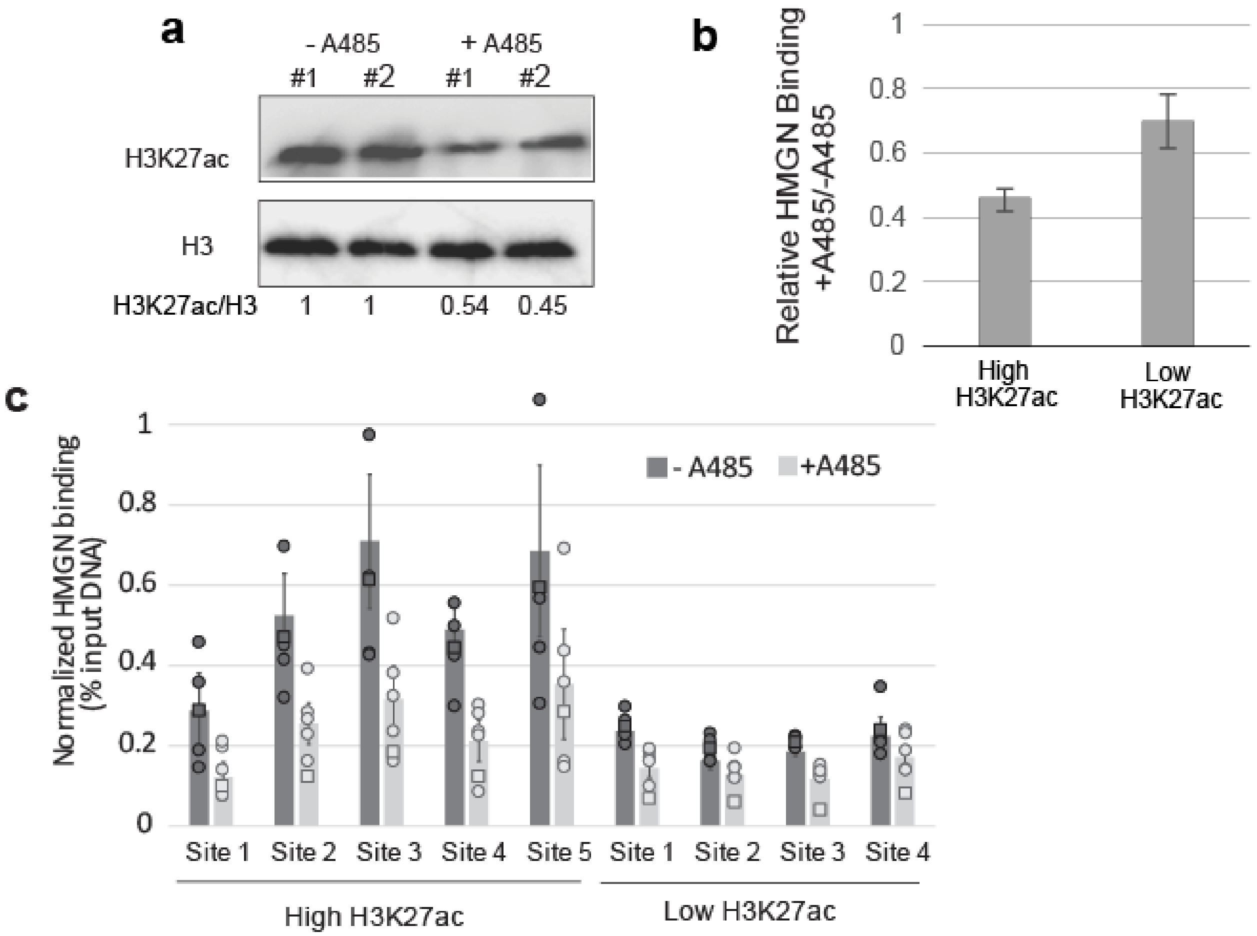

Supplementary Figure 5. Reduced H3K27ac decreases HMGN binding. (a) Western analysis of control and A-485 treated MEFs**. (b)** Binding of HMGN1 to selected sites in MEFs. **(c)** Average loss of HMGN1 binding in A-485 treated MEFs**.** Site co-ordinates and primer sequences are listed in Supplementary files, supplementary methods.

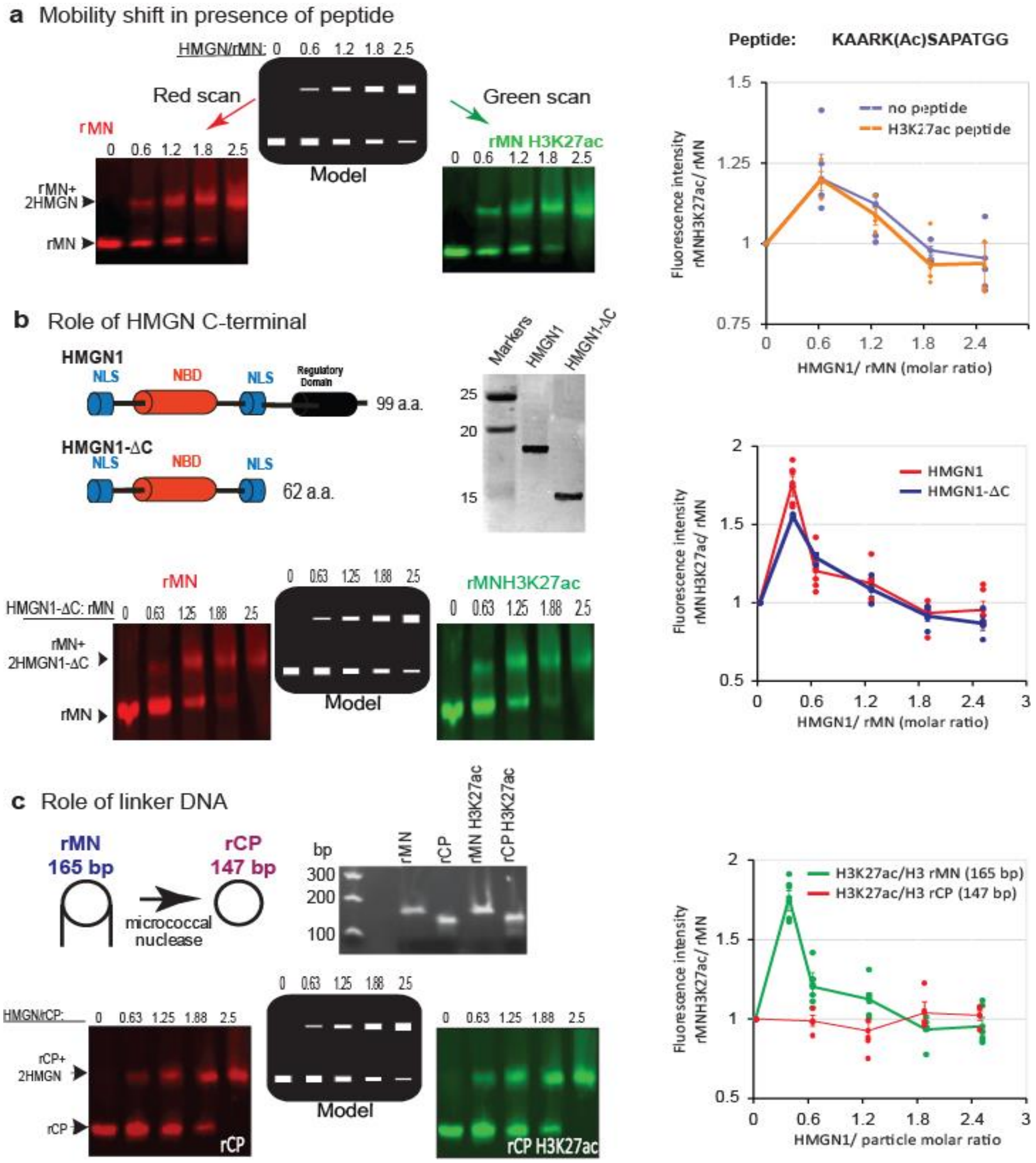

Supplementary Figure 6. Determinants of preferential binding of HMGN to MNH3K27ac. (a) HMGN1 does not bind specifically to the H3K27ac residue**.** Gel images of two-color mobility shift assays done in the presence of 1000-fold molar excess of a peptide spanning residues 23-34 of H3 in which residue K27 was acetylated. Sequence of the peptide and quantification of the gels is shown on the right. **(b)** The C-terminal domain of HMGN1 is not necessary for preferential binding to rMNH3K27ac. Shown is a schematic diagram of full length and of the C terminal deletion mutant of HMGN1 (HMGN1-ΔC) and polyacrylamide gels of purified HMGN1 and the deletion mutant; right, quantification of mobility shift of acetylated and non-acetylated rMN with the HMGN1-ΔC particles. (**c**) Linker DNA is necessary for the preferential binding of HMGN to acetylated nucleosomes. Left: Schematic diagram of rMN and nucleosome core particle (rCP); center: gels of DNA from acetylated and non-acetylated rMN and rCP particles; left bottom: images of two-color mobility shifts. Right, quantification of two-color gel mobility shift assays of HMGN1 with H3K27ac and nonacetylated rMN and rCP. Error bars show standard deviation, n≥3

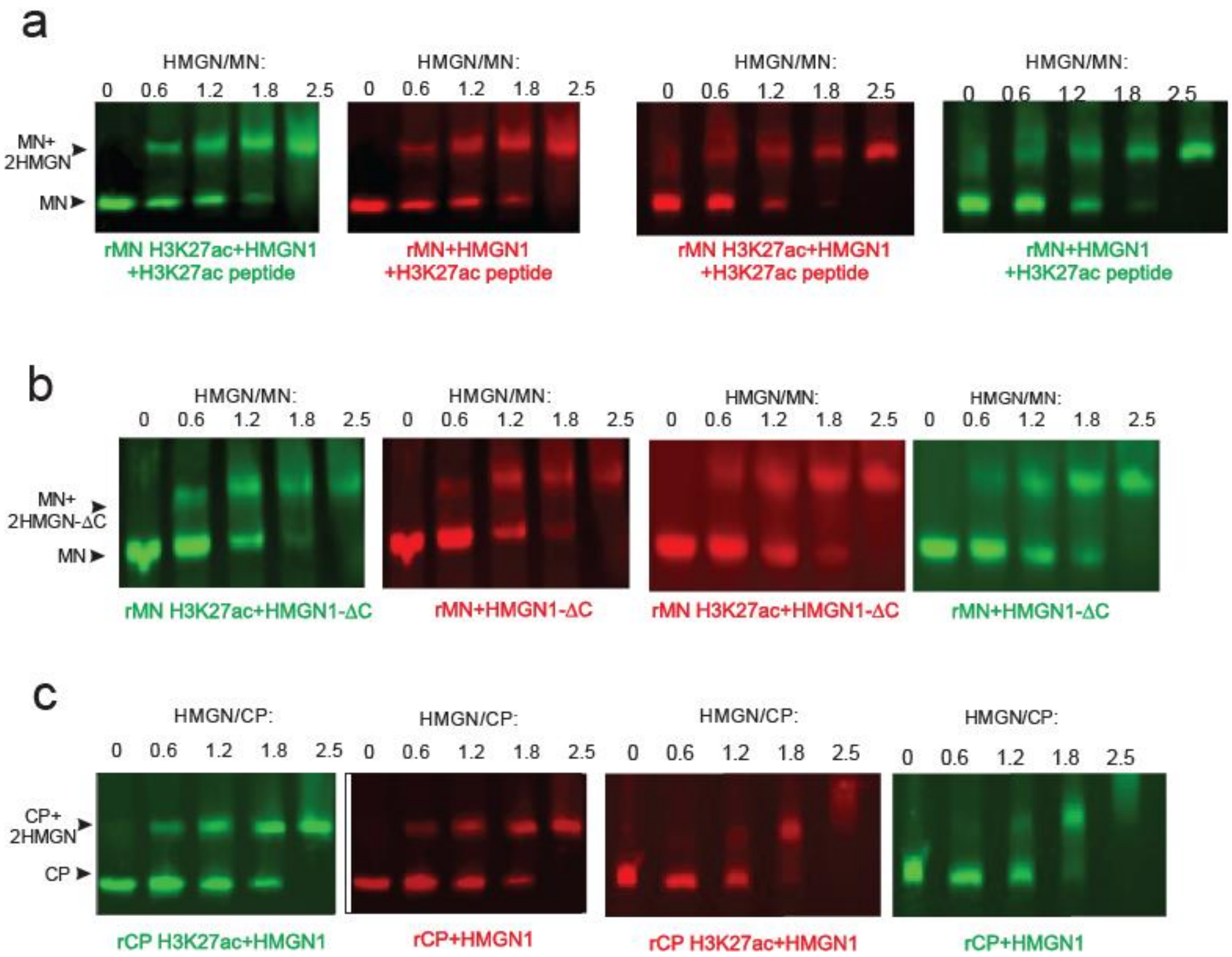

Supplementary Figure 7. Original two-color scans of nucleosome mobility shift assay. Original two-color scans of nucleosome mobility shift assays shown in Supplementary Fig. 6. A mix of equal amounts of fluorescently Alexa 488 labeled rMN (green) and Alexa 647 labeled rMNH3K27ac (red) were incubated with or without K27-acetylated mimicking peptide **(a)** with various amounts of HMGN and its C-termini deletion mutant (**b).** Same procedure has been repeated with core particles instead of nucleosomes **(c)**. The mixture was fractionated on native polyacrylamide gels, and the gels were scanned to visualize and quantify either the red or green fluorescence. The procedure was then repeated with swapped colors.

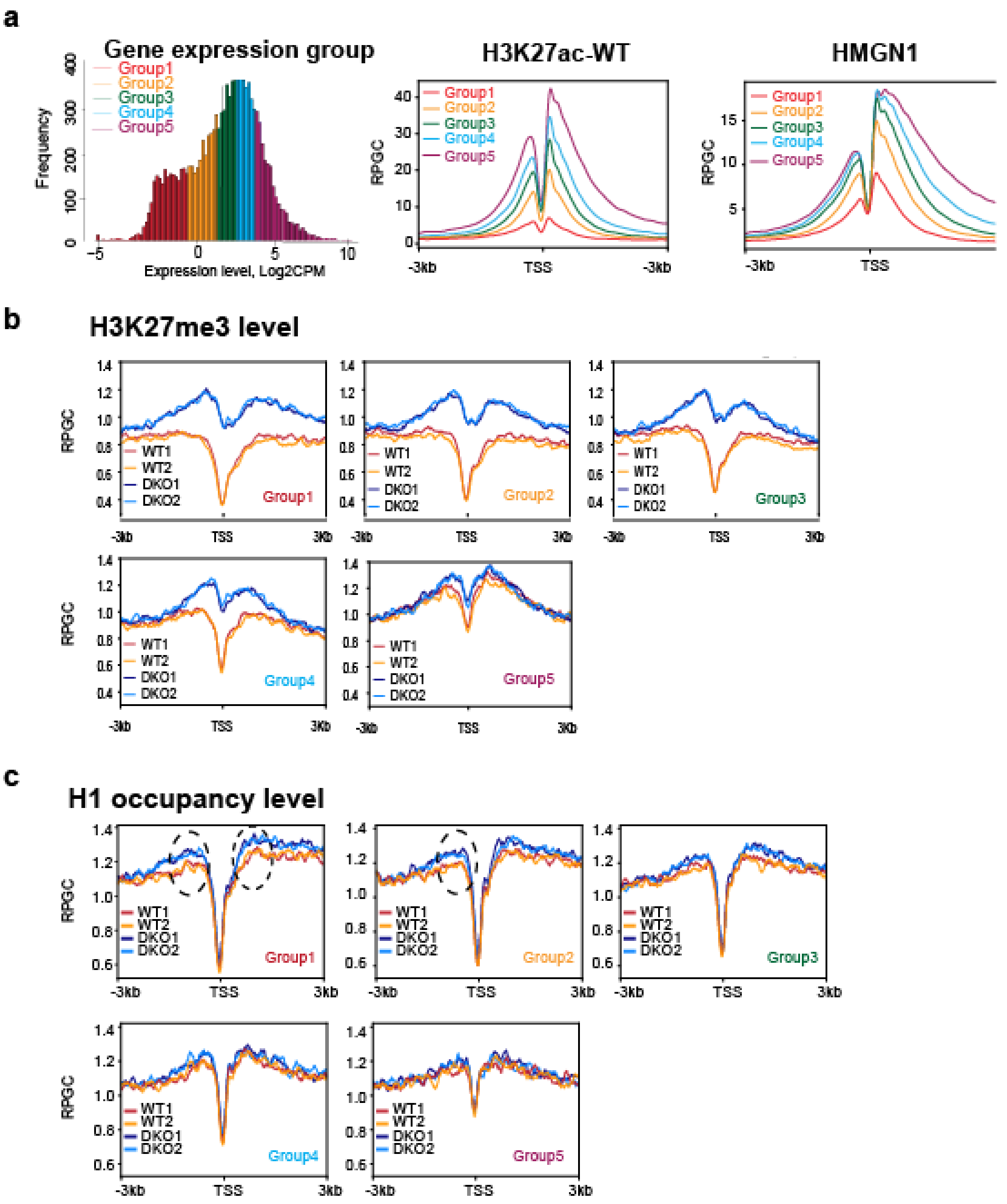

Supplementary Figure 8. HMGN mediated epigenetic changes correlate with the magnitude of gene expression. (a) Genes were sorted into 5 tiers according to expression levels (left). Both the H3K27ac levels (center) and the HMGN occupancy (right) at TSS correlate positively with gene expression levels. **(b)** H3K27me3 levels at the TSS of WT and DKO MEFs sorted by transcription level tier (as defined in panel A). Note that the largest difference between WT and DKO is seen at the most highly expressed genes, which have the highest content of H3K27ac and HMGN. **(c)** H1 occupancy levels at the TSS of WT and DKO MEFs sorted by transcription level tier. Note that the largest difference between WT and DKO is seen at the most highly expressed genes. All ChIP analyses done with 2 biological replicates.

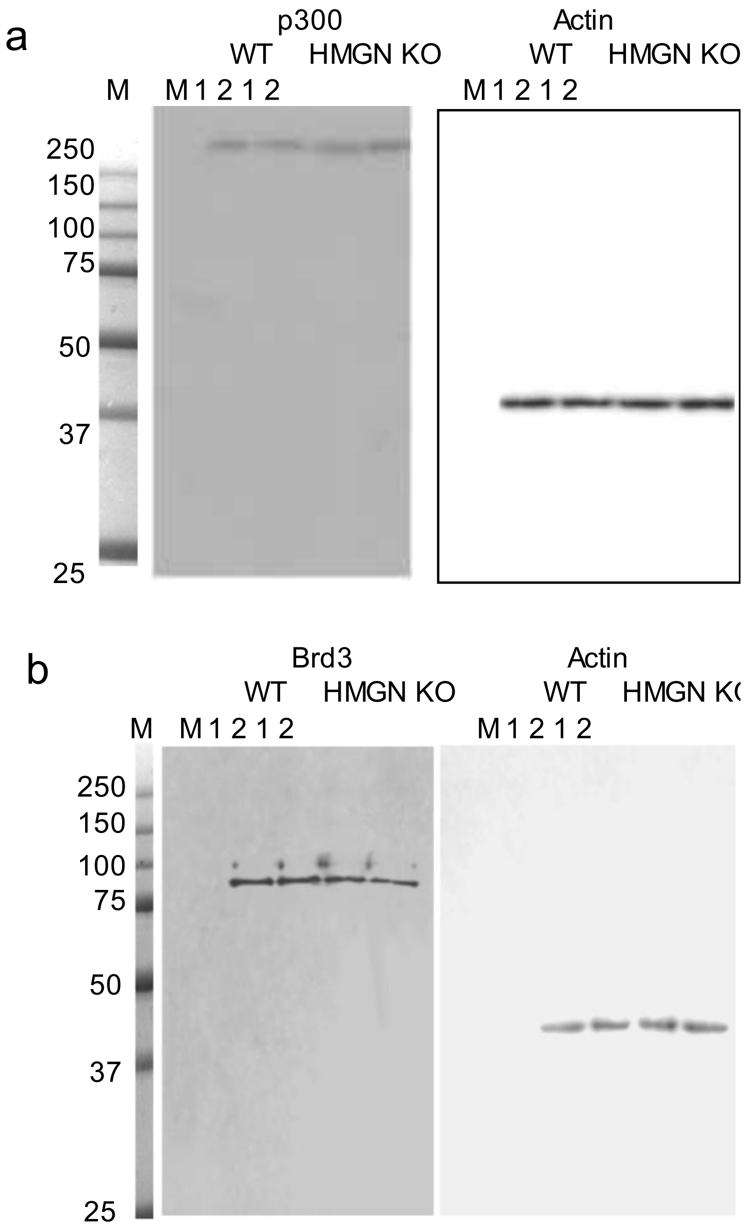

Supplementary Figure 9. Uncropped Western blot image with the size markers for Fig. 4a, 4e. Western blotting of whole cell extracts in two replicates of WT and DKO MEFs (antibodies used: p300**(a)**, Brd3 (**b**) and actin (a,b).

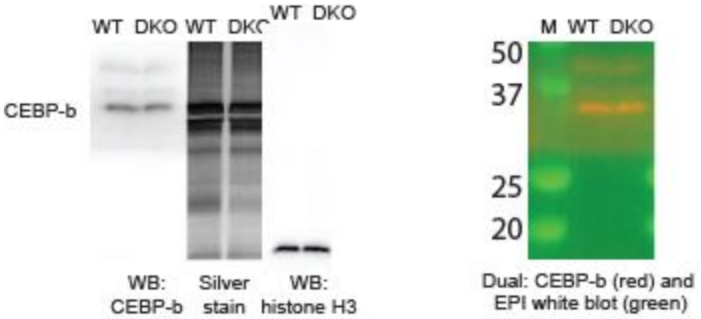

Supplementary Figure 10. Uncropped Western blot image with the size markers for Fig. 5a. Western blotting and Silver staining of whole cell extracts in WT and DKO MEFs (antibodies used: CEBPB and histone H3); two-mode blot scanning (overlay of pre-stained molecular weight markers and CEBPB Western ECL blot)

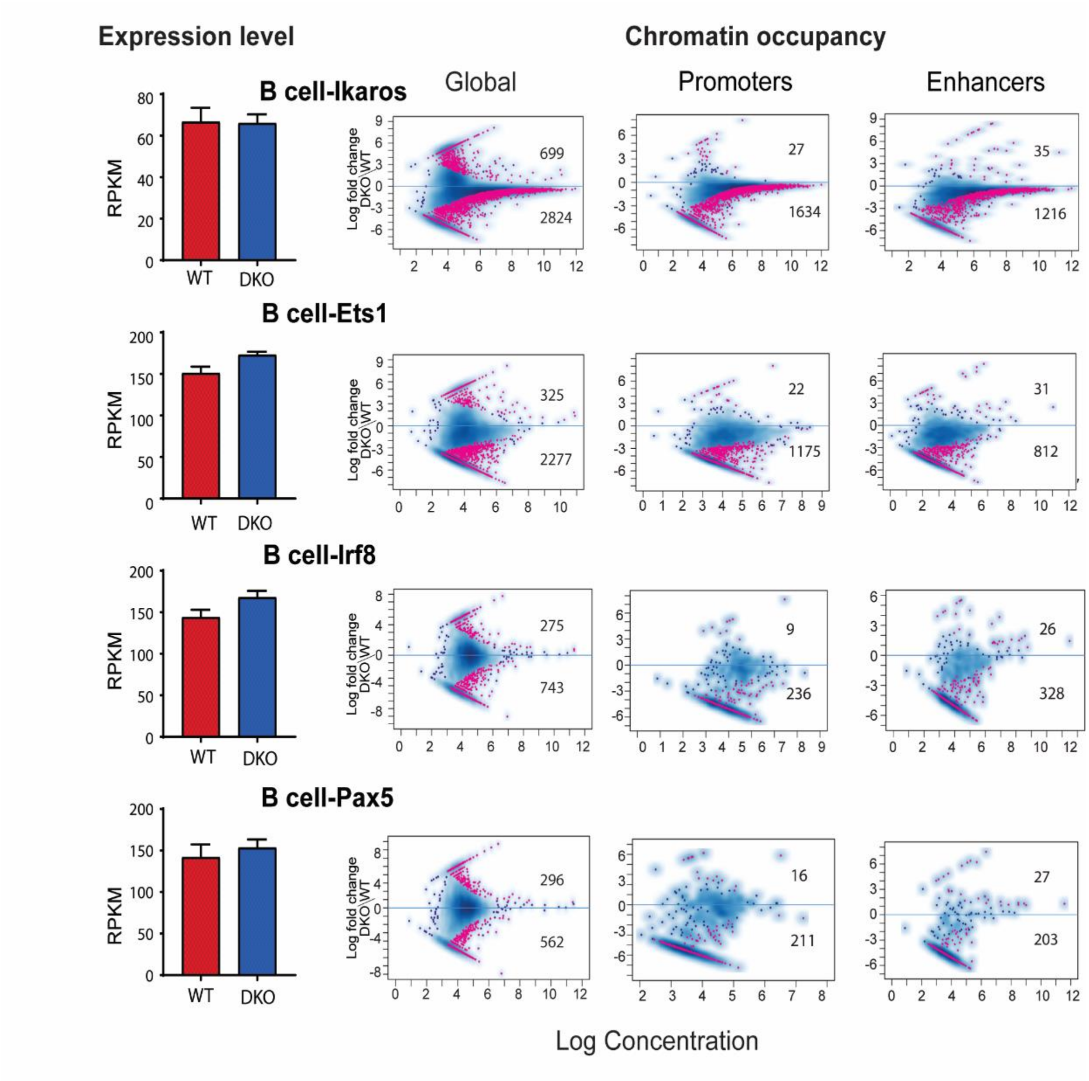

Supplementary Figure 11. Altered chromatin occupancy of transcription factors in DKO resting B cells. Bar graphs show transcript levels determined by RNA seq analysis. MA plots show differences in TF chromatin binding between DKO and WT cells. Statistically significant differences (FDR<0.05) are shown in red. Blue dots and blue density cloud represents all points corresponding to the non-changing regions. All data from 2 biological replicates.

Supplementary Table 1. The levels of histone H3 modifications in HMGN-bound nucleosomes

			H3 Modified/H3			
**Substrate**	**Method**	**Para-meter measured**	H3	H3K27ac	H3K9ac	H3K27me3
ON MEF	ChIP(anti-HMGN1)-WB	B/I	1	2.7		
MN RBC	GMSA (HMGN1) –WB	HA/LA	1	2.2		
MN RBC	GMSA (HMGN1) – dWB	HA/LA	1	2.3	1.1	0.9
MN RBC	GMSA (HMGN2) - dWB	HA/LA	1	1.6	1	0.9
ON MEF	ChIP(anti-HMGN1)-dWB	B/I	1	3.1	0.8	0.9
ON MEF	ChIP(anti-HMGN2)-dWB	B/I	1	3.3	1	0.9
rMN	dual-color GMSA (HMGN1)	red/green	1	1.7		1.1
rMN	dual-color GMSA (HMGN2)	red/green	1	1.6		1.1

Legend: MN: mononucleosomes, ON: oligonucleosomes, RBC: red blood cells, MEF: mouse embryonic fibroblasts, WB: Western blotting, dWB: dot Western blotting, GMSA: gel mobility shift assay, HA/LA: high affinity MN/ low affinity MN (see Fig. 2c), B/I: bound/input ratio.

Supplementary Materials and Methods

Cell culture. MEF cells were cultured in DMEM with 10% FBS, at 37 °C with 5% CO_2. To inhibit acetylation, the cells were cultured in 1µM A-485 (MedChemExpress #HY-107455) for 3 hrs.

Western blot analysis. Protein extracts were separated by SDS-15% PAGE gel electrophoresis and transferred to PVDF membranes using Invitrogen iBlot 2 system. Blocked with 5% milk, the membranes were incubated with antibodies and developed using SuperSignal WestPico PLUS (ThermoFisher #34577) enhanced chemiluminescence detection system. BioRad's ChemiDoc MP Imaging system and ImageLab Imaging Software v6.0.1 was used for visualization and quantitation.

Antibodies.

Anti-histone H3 (in-house)

Anti-Histone H3 (acetyl K27) antibody (Abcam, ab4729)

Real-time PCR. Quantitative real-time qPCR (qRT-PCR) was performed under adjusted cycling variables on an ABI 7900HT instrument using ThermoFisher SYBR Green Mastermix. Cycle threshold (C_T) values for each HMGN1-ChIP-DNA, were normalized to Input DNA (Percent Input Method), (dCt value) and converted to abundance value using Levak equation. Typically, 0.1% of starting chromatin was used as an input. Every PCR pair series with either with double Tm, extra additional band on agarose gel or No DNA positive signal were filtered out.

Chromatin immunoprecipitation (ChIP) assay. ChIP chromatin was prepared using a commercially available ChIP-IT kit (Active Motif) according to manufacturer's instructions. Beads with bound immunocomplexes were washed, eluted, and reverse cross-linked at 65°C overnight, subjected for RNase (final; 40 µg/ml) and proteinase K (Final; 100 µg/ ml) treatment and DNA were extracted by phenol–chloroform method. Input was prepared from 0.1% of the sonicated chromatin.

Primer selection. The primers were selected by Primer3-Blast tool using mouse genome assembly mm10, at 70-200 bp PCR product size, with each primer pair Specificity filter.

Primer coordinates

Regulatory site 1, Forward	TGCTGAAATTGCAAGGCTGA	chr3:90,614,202-90,614,221
Regulatory site 1, Reverse	CCCGGAAGTCATCTCAACGG	chr3:90,614,330-90,614,349
Regulatory site 2, Forward	CACAGCAGTGACAGGTCCAT	chr11:86,571,336-86,571,355
Regulatory site 2, Reverse	CTGCAGTACTGAGCCAGCAT	chr11:86,571,453-86,571,472
Regulatory site 3, Forward	CAAGAGCAGGAAGAGGGCAA	chr1:171,262,633-171,262,652
Regulatory site 3, Reverse	CTTCTTCAGGGGACACACCC	chr1:171,262,753-171,262,772
Regulatory site 4, Forward	TATGCTCCCGAAAGCAAGCA	chr7:19,816,915-19,816,934
Regulatory site 4, Reverse	CCCAAGTGGTCCCTCTCCTA	chr7:19,816,790-19,816,809
Regulatory site 5, Forward	ATGCACCTACTGCTGGCTTT	chr8:36,265,067-36,265,086
Regulatory site 5, Reverse	TGTTCAGCCACCACCCTAAG	chr8:36,265,139-36,265,158
Gene desert 1 on chromosome 6		(Active Motif, #71011)
Gene desert 2 on chromosome 17		(Active Motif, #71012)
Gene desert 3, Forward	GTCTCTGCTCCTGCTTAGCC	chr15:60,374,202-60,374,221
Gene desert 3, Reverse	GGGTATCCAGGCAGTGTAGC	chr15:60,374,341-60,374,360
Gene desert 4, Forward	TCTGAACTGCAACCAAAGTCCT	chr4:79,860,048-79,860,069
Gene desert 4, Reverse	GCACTGCTCTTTGTAGGTTTCA	chr4:79,860,194-79,860,215
