# Supplementary material for: H3K27ac nucleosomes facilitate HMGN localization at regulatory sites to modulate chromatin binding of transcription factors
Source: Commun Biol. 2022 Feb 23;5:159. doi: 10.1038/s42003-022-03099-0 (PMC8866397; doi:10.1038/s42003-022-03099-0)
Supplement: Supplementary file 2 — Description of Additional Supplementary Files [file 42003_2022_3099_MOESM2_ESM.pdf]

Description of Additional Supplementary Files

File name: Supplementary Data 1
Description: Source data for Figures 2G, Supplementary Figures 6a,b,c.
