# Supplementary material for: H3K27ac nucleosomes facilitate HMGN localization at regulatory sites to modulate chromatin binding of transcription factors
Source: Commun Biol. 2022 Feb 23;5:159. doi: 10.1038/s42003-022-03099-0 (PMC8866397; doi:10.1038/s42003-022-03099-0)
Supplement: Supplementary file 4 — Reporting Summary [file 42003_2022_3099_MOESM4_ESM.pdf]

nature portfolio

Corresponding author(s): Michael Bustin
COMMSBIO-21-1355B

Last updated by author(s): Jan 12, 2022

Reporting Summary

Nature Portfolio wishes to improve the reproducibility of the work that we publish. This form provides structure for consistency and transparency in reporting. For further information on Nature Portfolio policies, see our Editorial Policies and the Editorial Policy Checklist.

Statistics

For all statistical analyses, confirm that the following items are present in the figure legend, table legend, main text, or Methods section.

n/a	Confirmed	
☐	☒	The exact sample size (*n*) for each experimental group/condition, given as a discrete number and unit of measurement
☐	☒	A statement on whether measurements were taken from distinct samples or whether the same sample was measured repeatedly
☐	☒	The statistical test(s) used AND whether they are one- or two-sided<br>*Only common tests should be described solely by name; describe more complex techniques in the Methods section.*
☐	☒	A description of all covariates tested
☐	☒	A description of any assumptions or corrections, such as tests of normality and adjustment for multiple comparisons
☐	☒	A full description of the statistical parameters including central tendency (e.g. means) or other basic estimates (e.g. regression coefficient) AND variation (e.g. standard deviation) or associated estimates of uncertainty (e.g. confidence intervals)
☐	☒	For null hypothesis testing, the test statistic (e.g. *F*, *t*, *r*) with confidence intervals, effect sizes, degrees of freedom and *P* value noted<br>*Give P values as exact values whenever suitable.*
☐	☒	For Bayesian analysis, information on the choice of priors and Markov chain Monte Carlo settings
☐	☒	For hierarchical and complex designs, identification of the appropriate level for tests and full reporting of outcomes
☐	☒	Estimates of effect sizes (e.g. Cohen's *d*, Pearson's *r*), indicating how they were calculated

Our web collection on statistics for biologists contains articles on many of the points above.

Software and code

Policy information about availability of computer code

Data collection	ChIP-Seq: Illumina NextSeq 500 (GPL19057) and HiSeq 2000 (GPL13112); WB: BioRad ChemiDoc MP
Data analysis	ChIP-Seq software: MACS2 v. 2.1.1.20160309, CutAdapt v.1.16, FastQC 0.11.5, Kraken v1.1, FastQscreen v.0.9.3, Picard v. 2.17.11, SICER v. 1.1, DeepTools v. 3.0.1; WB: BioRad Image Lab Touch 6.1 and QuantityOne 1-D

For manuscripts utilizing custom algorithms or software that are central to the research but not yet described in published literature, software must be made available to editors and reviewers. We strongly encourage code deposition in a community repository (e.g. GitHub). See the Nature Portfolio guidelines for submitting code & software for further information.

Data

Policy information about availability of data

All manuscripts must include a data availability statement. This statement should provide the following information, where applicable:

- Accession codes, unique identifiers, or web links for publicly available datasets
- A description of any restrictions on data availability
- For clinical datasets or third party data, please ensure that the statement adheres to our policy

All Sequences generated: GSE156697, other ChIP-Seq: H3K9ac (GSM2417092), H3K4me1 (GSM2629668), H3K27ac (SRP154652) H3K122ac (SRR3144856), HMGNs (SRP154652), H3K27me3 (SRP068453) ;MEF cells: H3K9ac (GSM1979773), H3K4me1 (GSM3272827), HMGNs(SRP154652); rB cells-H3K27ac(SRP154652), HMGNs(SRP154652). H3K27me3(GSM2184272)

Field-specific reporting

Please select the one below that is the best fit for your research. If you are not sure, read the appropriate sections before making your selection.

☒ Life sciences ☐ Behavioural & social sciences ☐ Ecological, evolutionary & environmental sciences

For a reference copy of the document with all sections, see nature.com/documents/nr-reporting-summary-flat.pdf

Life sciences study design

All studies must disclose on these points even when the disclosure is negative.

Sample size	For ChIP-Seq experiments, n=3 was chosen as the minimal biological replicate number, and sample size was determined by the number of sequence reads (ENCODE and other recommendations). Internal control - genomic, "input" DNA.
Data exclusions	No data excluded
Replication	All replication attempts were successful. Cells were obtained from matched groups of mice.
Randomization	For all ChIP-Seq data and WB data here was no need for randomization
Blinding	For all ChIP-Seq data and WB data here was no need for blinding

Reporting for specific materials, systems and methods

We require information from authors about some types of materials, experimental systems and methods used in many studies. Here, indicate whether each material, system or method listed is relevant to your study. If you are not sure if a list item applies to your research, read the appropriate section before selecting a response.

Materials & experimental systems

n/a	Involved in the study	
☐	☒	Antibodies
☐	☒	Eukaryotic cell lines
☒	☐	Palaeontology and archaeology
☒	☐	Animals and other organisms
☒	☐	Human research participants
☒	☐	Clinical data
☒	☐	Dual use research of concern

Methods

n/a	Involved in the study	
☐	☒	ChIP-seq
☒	☐	Flow cytometry
☒	☐	MRI-based neuroimaging

Antibodies

Antibodies used	Rabbit polyclonal to H1, HMGN1, HMGN2 and H3 were from our laboratory, anti H3K27ac (Abcam#ab4729), anti H3K27me3 (Abcam#ab6002), monoclonal anti H1(Milipore-Sigma #05-457), anti CEBPB (Abcam#ab32358), Anti-Brd3 (Active Motif #61489), Anti-Brd4 (Bethyl Laboratories #A301-985A100), Anti-CEBPB (Abcam #ab32358), Anti-CTCF (EMD Millipore #07-729), Anti-Ets1 (Active Motif #39580), Anti-Ikaros (Active Motif #39355), Anti-Irf8 (Bethyl Laboratories #A304-027A), Anti-Klf4 (Abcam #106629), Anti-Nanog (Active Motif #61419), Anti-Oct4 (Abcam #ab19857), Anti-p300 (Active Motif #61401), Anti-Pax5 (Abcam #183575), Anti-Sox2 (Abcam #97959).
Validation	All commercial antibodies were validated for the method used in the study: either ChIP-Seq or WB grade. All in-house antibodies were validated in corresponding reference PMID: 791675

Eukaryotic cell lines

Policy information about cell lines

Cell line source(s)	Primary cell cultures from wild-type and knock-out HMGN1/ HMGN2: MEFs from mouse embryos at dpc13.5 and mouse ESCs from pre-implantation stage embryos
Authentication	None of the cell lines have been authenticated.
Mycoplasma contamination	Cell lines were tested for mycoplasma - no contamination
Commonly misidentified lines (See ICLAC register)	No commonly misidentified cell lines were used.

ChIP-seq

Data deposition

☒ Confirm that both raw and final processed data have been deposited in a public database such as GEO.

☒ Confirm that you have deposited or provided access to graph files (e.g. BED files) for the called peaks.

Data access links<br>*May remain private before publication.*	SRA selector run: BioProject PRJNA658791; GEO Accession: GSE156697
Files in database submission	76 files: GSM4742496-GSM4742571
Genome browser session<br>(e.g. UCSC)	SRA selector run: BioProject PRJNA658791; GEO Accession: GSE156697

Methodology

Replicates	For ChIP-Seq experiments, the minimal biological replicate number was 3
Sequencing depth	For the various samples the number of trimmed reads, successfully mapped to the mouse genome, ranged from 12 to 74 million per sample, with an average of 27 million reads with over 80% of trimmed, non-duplicated reads mapped to the genome.
Antibodies	Rabbit polyclonal anti-histone H1, anti-HMGN1, anti-hHMGN2 and anti-histone H3 w(in-house), anti H3K27ac (Abcam#ab4729), anti H3K27me3 (Abcam#ab6002), monoclonal anti H1(Milipore-Sigma #05-457), anti CEBPB (Abcam#ab32358), Anti-Brd3 (Active Motif #61489), Anti-Brd4 (Bethyl Laboratories #A301-985A100), Anti-CEBPB (Abcam #ab32358), Anti-CTCF (EMD Millipore #07-729), Anti-Ets1 (Active Motif #39580), Anti-Ikaros (Active Motif #39355), Anti-Irf8 (Bethyl Laboratories #A304-027A), Anti-Klf4 (Abcam #106629), Anti-Nanog (Active Motif #61419), Anti-Oct4 (Abcam #ab19857), Anti-p300 (Active Motif #61401), Anti-Pax5 (Abcam #183575), Anti-Sox2 (Abcam #97959).
Peak calling parameters	Peak calling was done by MACS2 v. 2.1.1.20160309 with the default settings, and by SICER v. 1.1 with the following parameters: window size 300, gap size 600, FDR < 1e-2, effective genome size 0.75.
Data quality	QC analysis has been done by quantification of number of reads in peaks, low background, sequencing depth according to ChIP-seq guidelines of the ENCODE and modENCODE consortia
Software	MACS2 v. 2.1.1.20160309, CutAdapt v.1.16, FastQC 0.11.5, Kraken v1.1, FastQscreen v.0.9.3, Picard v. 2.17.11, SICER v. 1.1, DeepTools v. 3.0.1; R (ver. 3.6) computing environment and visualized with IGV_2.6.2.
